# Combining independent component analysis and source localization for improving spatial sampling of stereoelectroencephalography in epilepsy

**DOI:** 10.1038/s41598-024-54359-4

**Published:** 2024-02-19

**Authors:** Samuel Medina Villalon, Julia Makhalova, Victor J. López-Madrona, Elodie Garnier, Jean-Michel Badier, Fabrice Bartolomei, Christian G. Bénar

**Affiliations:** 1 APHM, Timone Hospital, Epileptology and Cerebral Rhythmology, Marseille, France; 2 Aix Marseille Univ, INSERM, INS, Inst Neurosci Syst, Marseille, France

**Keywords:** Epilepsy, Seizure-onset zone, icEEG, ICA, Source localization, Biomarkers, Neurology, Mathematics and computing

## Abstract

Stereoelectroencephalography is a powerful intracerebral EEG recording method for the presurgical evaluation of epilepsy. It consists in implanting depth electrodes in the patient’s brain to record electrical activity and map the epileptogenic zone, which should be resected to render the patient seizure-free. Stereoelectroencephalography has high spatial accuracy and signal-to-noise ratio but remains limited in the coverage of the explored brain regions. Thus, the implantation might provide a suboptimal sampling of epileptogenic regions. We investigate the potential of improving a suboptimal stereoelectroencephalography recording by performing source localization on stereoelectroencephalography signals. We propose combining independent component analysis, connectivity measures to identify components of interest, and distributed source modelling. This approach was tested on two patients with two implantations each, the first failing to characterize the epileptogenic zone and the second giving a better diagnosis. We demonstrate that ictal and interictal source localization performed on the first stereoelectroencephalography recordings matches the findings of the second stereo-EEG exploration. Our findings suggest that independent component analysis followed by source localization on the topographies of interest is a promising method for retrieving the epileptogenic zone in case of suboptimal implantation.

## Introduction

Stereoelectroencephalography (SEEG) is an established technique of intracerebral EEG recording for the presurgical evaluation of epilepsy. In the last 10 years, this technique has become the recognized gold standard of invasive exploration^1^. The implantation is guided by clinical hypotheses following a series of non-invasive investigations^2^. The main objective of SEEG is to define the epileptogenic zone network (EZN), characterized by distinct seizure-onset patterns, a spatial extent, and the timing of involvement of its different structures. The EZN can be quantified by estimating epileptogenicity at seizure onset, e.g. by the epileptogenicity index^3^ (EI), eventually complemented by other measures such as interictal spikes^4,5^ or connectivity^6,7^. SEEG allows measuring brain activities with millimeter topographic precision and millisecond temporal resolution, targeting equally well superficial or deep regions. The main limitation of SEEG is the sampling problem: the number of electrodes is limited, and the EZN can be missed or sub-optimally explored.

Source localization is commonly used in non-invasive recordings (EEG and magnetoencephalography, MEG) to localize physiological and pathological brain activity. This technique relies on models of the origin and propagation of the electromagnetic fields in the head volume, and mathematical tools for inferring the brain sources from surface measures (the so-called “inverse problem” of EEG/MEG). Recently, these techniques have been extended to SEEG signals^8–12^.

Independent component analysis (ICA) is a methodologically different technique that separates activities from multiple sources mixed at the sensor level. It is used in non-invasive recordings to remove artifacts^13^ or to extract activities of interest such as epileptic discharges or cognitive processes^14,15^. Each independent component comprises a time course and a spatial pattern (topography), and can be considered as a node of an epileptogenic network^16^. Source localization can be applied to each topography^17^ or a set of topographies^18^ to identify the origin of the epileptic activity.

We have recently demonstrated that combining ICA and source localization on SEEG signals potentially allows identifying sources that are remote from the sensors^10^. However, the use of source localization on SEEG signals is still scarce^8–12^, even though it has the potential to estimate brain activity of the regions that were not sampled and thus to extend the field of view of SEEG. In this work, we addressed the question of whether ICA-based source localization analysis on a first, undersampled SEEG can predict the results of a second SEEG exploration.

## Materials and methods

### Patients

Two patients who underwent two consecutive SEEG explorations were retrospectively selected. The first SEEG implantation resulted in a suboptimal mapping of the EZN requiring a second SEEG that provided a satisfactory localization of the EZN, followed by a successful surgical procedure in terms of seizure outcome. Informed consent was obtained from the Patient 2 and from the parents for the Patient 1, aged below 18 years. The study was approved by the Assistance Publique – Hôpitaux de Marseille (health data access portal registration number PADS L56C78). Recordings, interpretation and analysis of SEEG were performed following the French guidelines on stereoelectroencephalography^2^ and guidelines on SEEG analysis^19^.

**Figure 1:**
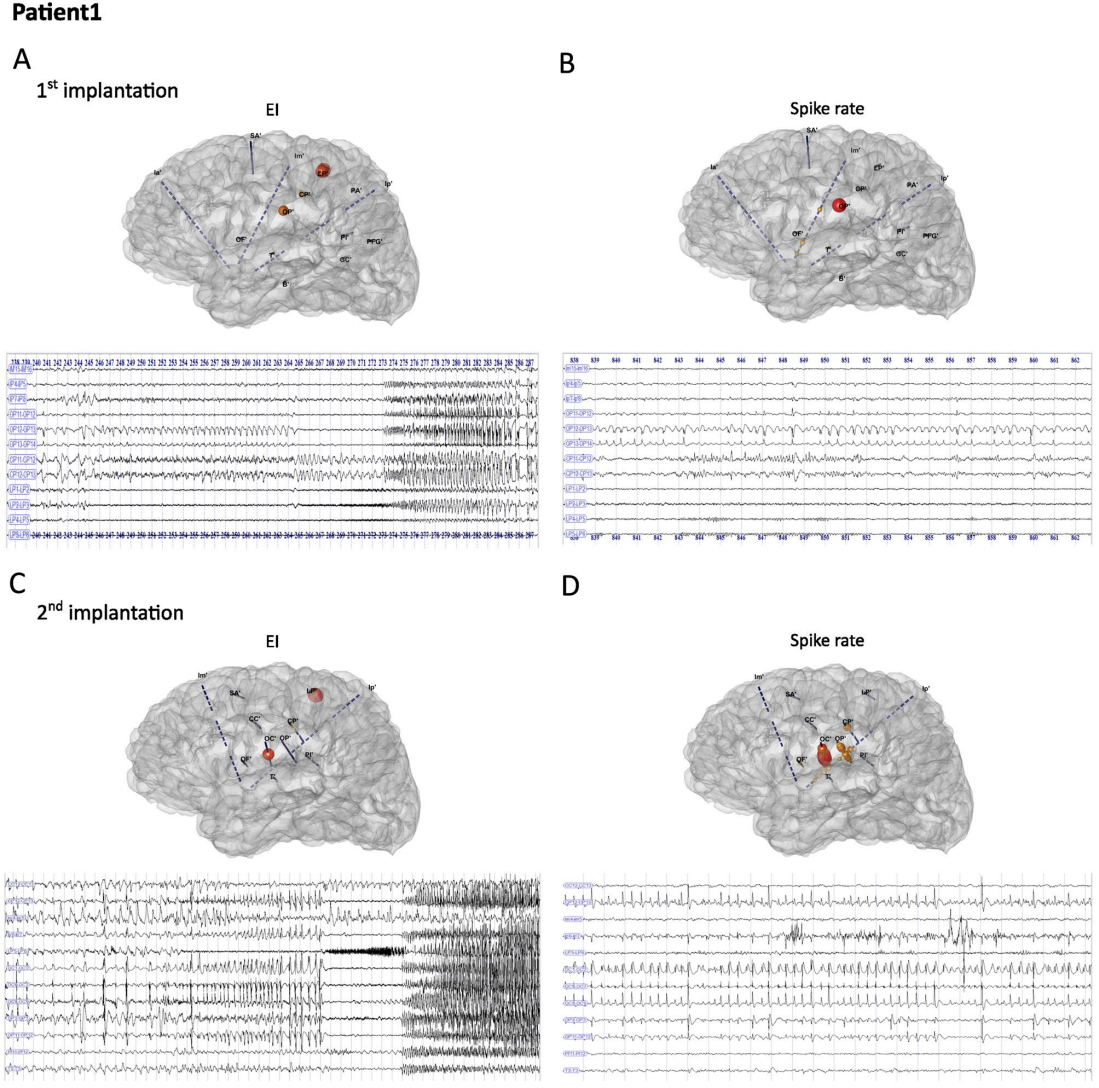
Ictal and interictal recordings from the two SEEG implantations in Patient 1. (**A**) Epileptogenicity index (EI, colored spheres on the patient’s 3d brain mesh) computed from the first SEEG ictal time series. SEEG recording of a habitual seizure that starts with a preictal spiking pattern visible over the left parietal operculum (OP'11–13), followed by the rapid discharge predominating over the ipsilateral motor cortex (LP’1–5). (**B**) Interictal data from the same SEEG. The maximal spike rates are represented as colored spheres on the patient’s 3d brain mesh. Interictal resting state time series showing rhythmic slow waves and sharp-waves over an altered background activity in the left parietal operculum (OP'12–14). (**C**) The second SEEG implantation shows that the maximum epileptogenicity is located more anteriorly in the operculo-insular region (the electrode OC' sampling the central operculum and the adjacent superior insula has been added in the second SEEG), based on visual and EI analysis of ictal signals. (**D**) Interictal recording showing subcontinuous rhythmic spike activity and the maximal interictal spike rates within these structures.

Patient 1 (16-year-old male) presented with mainly sleep-related seizures since age 7 years, characterized by tingling in the mouth followed by right facial and arm contraction, pedaling and right hemibody myoclonic jerks. Scalp video-EEG showed left centrotemporal spikes and left hemispheric ictal discharges. Brain MRI findings were normal; MEG showed several source localizations and IC in the left posterior perisylvien regions (posterior T1, planum temporale, parietal operculum), the right posterior T1 and the left precuneus. PET showed left operculo-insular hypometabolism. A first SEEG was performed, with 15 electrodes (Fig. 1A,B). Interictal activity was maximal in the left parietal operculum (OP'), with an electrical pattern suggestive of a focal cortical dysplasia (FCD, Fig. 1B). Seizures (Fig. 1A) were characterized by a preictal spiking pattern visible over the left parietal operculum but the rapid discharge that followed predominated over the ipsilateral motor cortex (LP’). Thermocoagulation targeting the OP' contacts was unsuccessful. Because of insufficient sampling of the posterior insulo-opercular region, a second SEEG was performed a few months later, with 11 left electrodes (Fig. 1C,D). Interictal recordings showed abundant subcontinuous rhythmic spike activity over the contacts exploring the left central operculum (OC'), the parietal operculum (OP') as well as the adjacent superior and posterior insula (OC'1–2; OP'1–2, Ip'1–7) (Fig. 1D). Seizures started by preictal spiking involving these structures and the postcentral gyrus (CP'), followed by a tonic fast discharge on the same contacts that simultaneously involved the motor cortex (LP’) (Fig. 1C). Usual seizures were reproduced by 1 Hz stimulations of the OC'4–7 contacts. Thermocoagulations were performed targeting the contacts OC'1–10, OP'1–12, CP'9–13. The patient remained completely seizure-free for one year. He then reported rare, sleep-related seizures limited to a left facial contraction (last follow-up at 3.5 years).

Patient 2 (20-year-old female) suffered from drug-resistant epilepsy since the age 6 months. She experienced daily seizures with jaw paraesthesia and contraction, difficulty to breath, followed by loss of consciousness and bilateral tonic posturing, sometimes with postictal left-hand deficit. Scalp EEG showed right fronto-central and temporal interictal spikes and ictal discharges. PET showed right temporo-perisylvian hypometabolism. A 3 T MRI disclosed an image consistent with FCD in the right precentral sulcus, close to the central operculum. A first SEEG with 11 electrodes was performed at age 12, with predominantly right temporal and operculo-insular exploration (Supplementary Fig. 1A,B). Subcontinuous spike activity was present in the right central operculum (OP), the insula (I2-4) and the precentral sulcus (I10-12, Supplementary Fig. 1B). Seizures started by preictal spiking followed by rapid discharge over these regions, also involving the frontal operculum and the dorsolateral premotor cortex (Supplementary Fig. 1A). A first surgery at age 13 removed the right insula and a part of opercular cortex near the OP electrode, without significant effect on seizures. A second SEEG was performed at age 19 (16 right and 3 left electrodes, Supplementary Fig. 1C,D) with the exploration of the remaining right operculo-perisylvian regions, and the remaining part of the dysplastic region. SEEG 2 showed maximal interictal activity within the right precentral lesional sulcus (OC3-5) (Supplementary Fig. 1D) and seizures beginning with preictal spiking followed by fast discharge in this region before rapid spread throughout the right fronto-parieto-opercular and the left insulo-opercular regions (Supplementary Fig. 1C). A usual seizure was triggered by 50 Hz stimulation of contacts OC2-3. Subsequent partial resection of the lesion area led to significant improvement of the epilepsy (rare motor seizures in the left hemiface, last follow-up at 2 years). Histopathological diagnosis was FCD type1.

### Data acquisition

MRI were acquired on a Siemens 3 T system according to a standardized presurgical assessment protocol that included 3D T1-weighted magnetization-prepared rapid gradient echo (MPRAGE) images. Intracerebral multiple contact electrodes (10–18 contacts with length 2 mm, diameter 0.8 mm, and 1.5 mm apart, Dixi) were placed stereotactically. Post-implantation CT was performed to check the electrodes positions and exclude intracranial bleeding. SEEG were recorded on a 256 channels Natus system, sampled at 1024 Hz and saved on a hard disk (16-bits resolution) using no digital filter. Two hardware filters were present in the acquisition procedure: a high pass filter (cut-off frequency equal to 1 Hz at − 3 dB) and an antialiasing low‐pass filter (cut-off at 340 Hz).

### Contact localization

Volumetric segmentation and cortical surface reconstruction from the patient’s MPRAGE data were obtained using the recon-all pipeline of the FreeSurfer (http://surfer.nmr.mgh.harvard.edu) software and were then imported into the Brainstorm software^20^. Co-registration of the MPRAGE with post-implantation CT images was performed, the SEEG electrodes contacts were automatically localized in patient-specific MRI space using GARDEL software (https://meg.univ-amu.fr/wiki/GARDEL:presentation).

### SEEG analysis and source localization

Different epileptogenicity markers were computed from the SEEG recordings. The epileptogenicity index (EI^3^) was computed to estimate the epileptogenicity at seizure onset. Interictal spikes were detected automatically from NREM sleep and resting-state recordings using Delphos software^21^. The maximal spike rates per channel were calculated.

We performed Independent Component Analysis (ICA) to disentangle different parts of the epileptogenic networks and summarize epileptic activities by a few components of interest^16^. ICA, based on the infomax algorithm^22^ as available in the AnyWave software (https://meg.univ-amu.fr/wiki/AnyWave), was computed on the entire ictal recordings and the interictal awake recordings (10–30 min) separately.

**Figure 2:**
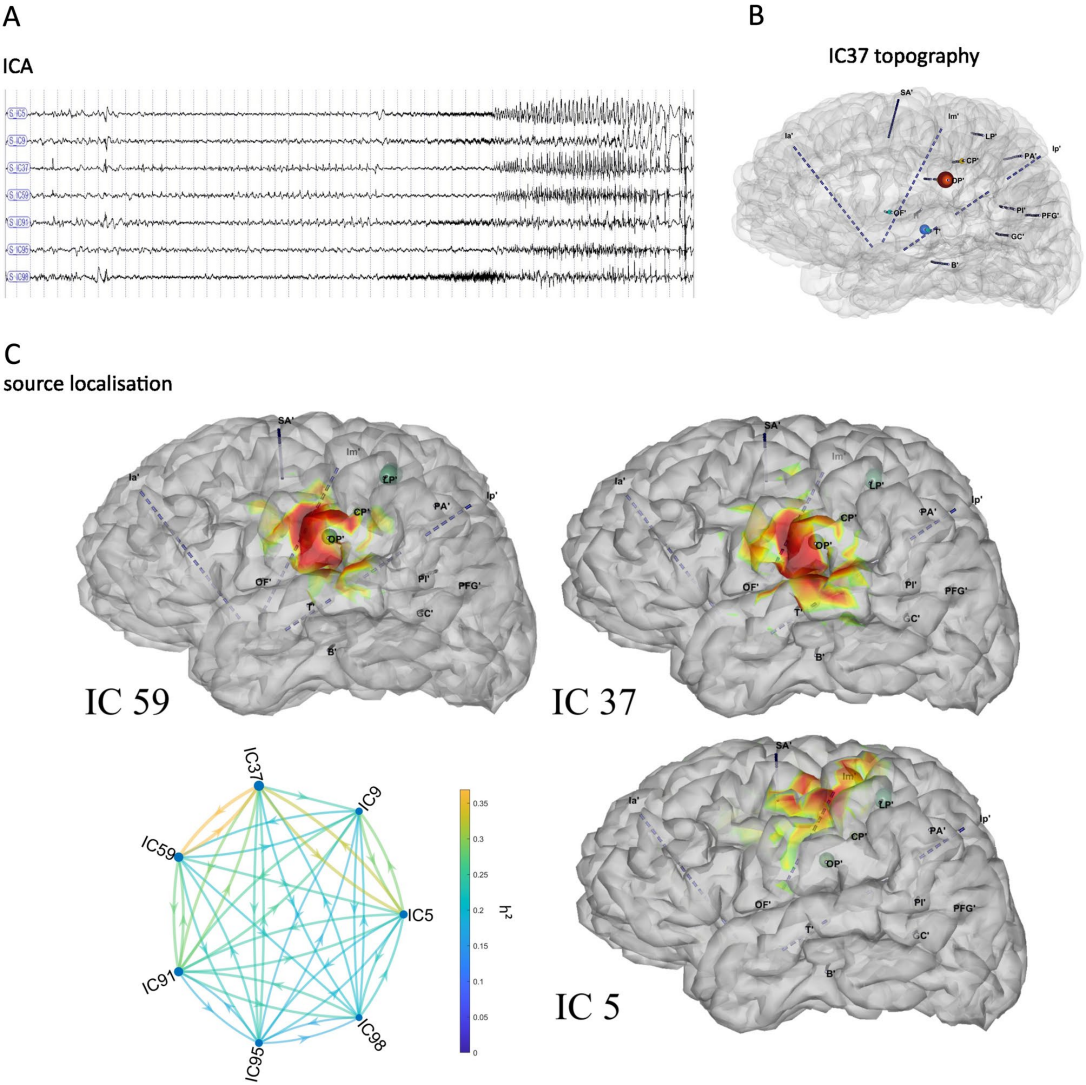
Illustration of the method of ICA followed by source localization on the ictal recording of Patient 1. (**A**) The ICA time series obtained from the monopolar SEEG recording of the first implantation. (**B**) Example of a topography (IC37) in the mesh of the patient1 represented by colored spheres and showing the contribution of OP’, T’, OF’. (**C**) Connectivity graph between the selected components. The links represent the mean h^2^, the directionality is given by the directionality index and the circles represent the strength of each node. Source localization of the three main components, IC59, IC37 and IC5, are shown as heatmaps on the patient’s 3d brain mesh (SLoreta map threshold = 50%).

The ICA disclosing epileptic components (interictal spikes/sharp-waves, preictal spiking and/or fast discharge) were selected visually by an experienced neurophysiologist (JM) (Fig. 2 top left). We then computed a directed connectivity graph between all pairs of selected components of the ictal or interictal dataset, respectively. In the graph, each node corresponds to the out-strength of a component (sum of all outgoing links comprising this node), and the links between the nodes to the connectivity value (Fig. 2.bottom left). For this measure, we used the nonlinear correlation coefficient h^2^, followed by the directionality index that combines delays and h^2^ asymmetry^7^. The components with high node out-strength and high connectivity values were considered network leaders^23^ and selected for source localization.

Source localization was performed on the component topographies^10^(Fig. 2 top right) using Brainstorm^20^. We first exported with GARDEL the electrodes coordinates in the MRI space^24^. Using in-house code, we exported ICA topographies to read them in Brainstorm. Forward model was computed using the OpenMEEG^25^ method. Finally, we used sLORETA for estimating the localization on the cortex of the topographies of interest (Fig. 2 bottom). We applied a threshold of 50% on the maps for visualization. For the noise covariance, we used the identity matrix. The whole pipeline (ICA followed by source localization) is further referred to as “ICA source localization”. Illustration of the process on interictal data of Patient 1 and ictal and interictal data of Patient 2 are shown in supplementary figures (Supplementary Figs. 2, 3 and 4 respectively).

## Results

ICA source localization was applied on each patient's ictal and interictal periods of the first SEEG. In Patient 1, the first SEEG (Fig. 1) insufficiently sampled the operculo-insular region. In particular, SEEG1 did not explore the left central operculum, which was found to be the core part of the EZN, expressing the maximal EI values on the second SEEG. However, both SEEG explorations showed high EI values in the left motor cortex. The Independent Components (IC) obtained on the ictal dataset and disclosing the greatest connectivity pattern are shown in Fig. 2. The connectivity graph on the epileptic components showed that IC59 and IC37 were leaders in the graph (the highest node out-strength and highest connectivity, Fig. 2C). The sources of these components were located within the left central and parietal operculum, anterior to the OP’ electrode. The IC5 as well as IC98 leaded by IC37 and IC59 in term of node strengths of the connectivity graph, were located in the left motor region (only the IC5 having a higher node strength, is illustrated), suggesting its involvement in the epileptogenic network as indicated by visual and EI analysis.

The ICA performed on the interictal dataset revealed several components with spikes (Supplementary Fig. 2). IC4 and IC8 were identified as leaders by the graph. Their localizations reproduced that of ictal ICA and corresponded to the lateral and inferior aspects of the left central and parietal operculum for IC4 and IC8, respectively. The IC1, receiving outgoing links from these leader components, was located in the left motor region.

In Patient 2, the first implantation explored the perilesional right frontal neocortex by the external contacts of the oblique electrode I, which were situated behind the bottom of the dysplastic precentral sulcus and did not sample its lateral aspect (Supplementary Fig. 1). The second SEEG better sampled the dysplastic region (the orthogonal electrode OC) and showed maximal EI values within this region. The SEEG1 ictal and interictal datasets with corresponding independent components are shown in Supplementary Figs. 3 and 4. The h^2^ graph on the ictal independent components (Supplementary Fig. 3C) showed that IC92, 100 and 97 were leaders in the network. The source localizations on IC92 and IC97 were focal and located in the bottom of the dysplastic precentral sulcus, anteriorly to the electrode I as well as in the insulo-opercular junction (IC97). The localization on IC100 was very widespread and not conclusive.

For the interictal activity (Supplementary Fig. 4), the components 31, 13 and 61 were identified as leaders in the network. The localizations of components 31 and 61 were in the bottom and the anterior aspect of the dysplastic sulcus, reproducing the ictal ICA; the component 13 was more spread.

**Figure 3:**
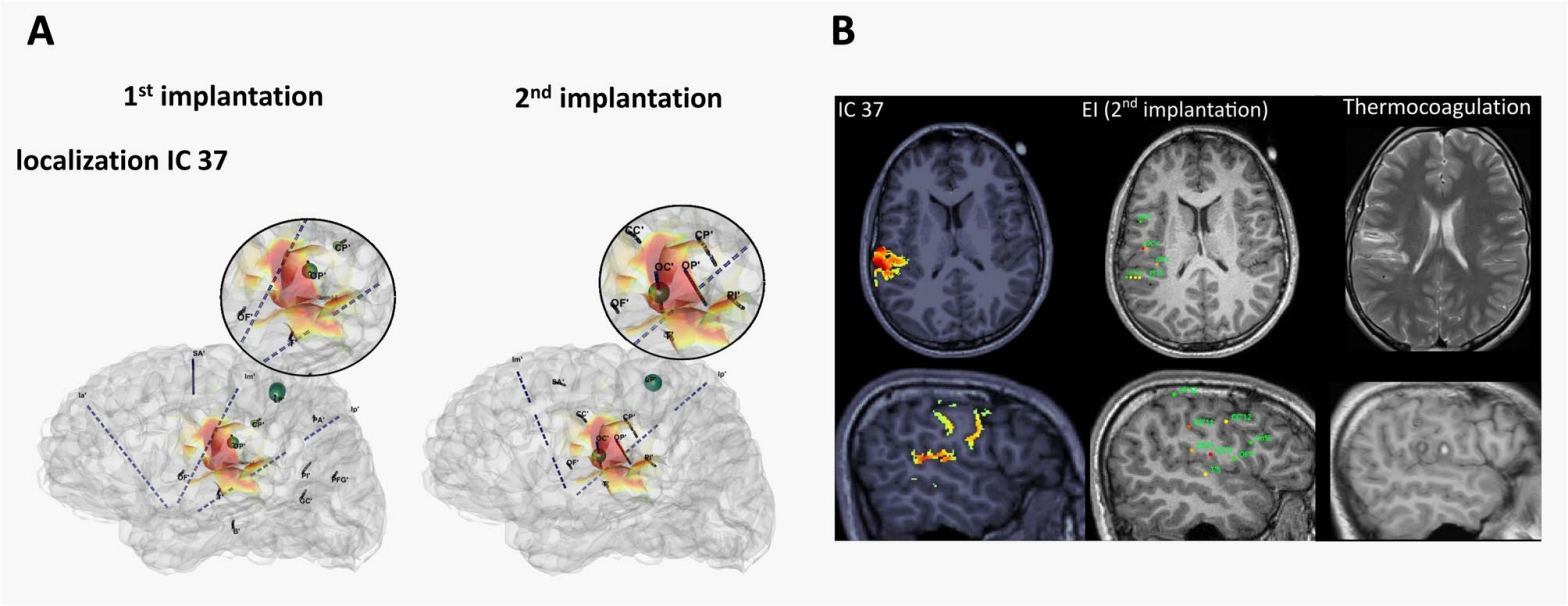
Correlations between ictal ICA source localization calculated on the first SEEG and epileptogenicity values estimated across the two successive SEEG explorations in Patient 1. (**A**) Ictal ICA source localization (heatmap) (SLoreta map threshold = 50%) of the main component of SEEG1 projected on the patient’s brain mesh with SEEG electrodes of the first (left) and the second (right) implantations. The maximal epileptogenicity index (EI) values are represented as shades of green spheres on the respective contacts. On the SEEG1, the EI shows maximal epileptogenicity within the left parietal operculum (OP’) and the left motor cortex (LP’). On the SEEG2, the maximal epileptogenicity is within the left central operculum (OC’, non-sampled by SEEG1) and the left motor cortex. Source localization of IC37 include the left central and parietal operculum and the left planum temporale. (**B**) ICA source localization of SEEG1 (left), the EI values of SEEG 2 (represented on the respective contacts according to a color scale from red, maximal epileptogenicity, to green, no epileptogenicity, middle) and the thermocoagulation lesions of SEEG2 (right) shown on the patient’s axial and sagittal MRI views. The peak of the IC37 matches with the central and parietal operculum, disclosing high epileptogenicity and targeted by thermocoagulation.

**Figure 4:**
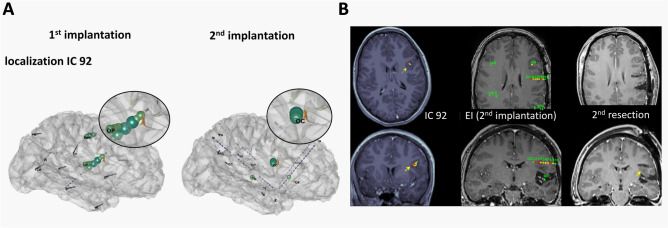
Correlations between ictal ICA source localization on the first SEEG and epileptogenicity values estimated across the two successive SEEG explorations in Patient 2. (**A**) Ictal ICA source localization (heatmap) (SLoreta map threshold = 50%) of the main component of SEEG1 and the maximal EI values (green spheres) projected on the patient’s brain mesh with SEEG electrodes of the first (left) and the second (right) implantations. Source localization of the IC92 is in the dysplastic right precentral sulcus and matches with the maximal EI values on the SEEG2 (electrode OC). (**B**) Patient’s axial and coronal MRI views showing ICA source localization of SEEG1 (left), the EI values of SEEG2 (represented on the respective contacts according to a color scale from red, maximal epileptogenicity, to green, no epileptogenicity), with the right insular resection performed after SEEG1 visible on the coronal slice (middle), and the resection of the perilesional opercular cortex after SEEG2 (right). The peak of the IC92 matches with the bottom of the dysplastic sulcus (yellow arrows) showing maximal epileptogenicity on SEEG2 and disconnected by the 2nd resection (removal could not be achieved due to the technical issues).

The topographic correlations between the ictal ICA source localization on SEEG1 and the epileptogenic regions quantified on the first and second SEEG are summarized in Fig. 3 (patient 1) and Fig. 4 (patient 2). For both patients, source localization of the leading independent components performed on the first, suboptimal implantation, overlapped with the contacts showing the maximal EI values and identified as the EZN on the second, successful implantation (OC’ in patient 1, Fig. 3A, and OC in patient 2, Fig. 4A). These localizations corresponded to the epileptogenic regions that were non- or undersampled by the first implantation (the central operculum in Patient 1; the dysplastic precentral sulcus in Patient 2). While the motor or premotor regions also disclosed high EI values (LP’ in Patient 1; SA in Patient 2), the corresponding independent components were not leading in the network.

## Discussion

This study is a proof of concept, showing that it is possible to define the EZN from an undersampled SEEG, using a combination of ICA and source localization^10^. The results of a second SEEG implantation served as ground truth to validate our strategy.

An essential advantage of ICA is that it helps extract epileptic activities of interest from background activities and provides associated fixed topographies that are not time-dependent. It avoids manual selection of epileptic spikes, averaging and source localization at specific time points of interest, which is time-consuming. Importantly, it also allows disentangling the different nodes of the epileptogenic networks^16^. As epileptic sources (corresponding each to a patch of activated cortex) are usually reasonably independent, each ICA topography is likely to correspond to a single source^26^. Nonetheless, there is no certainty in this regard, as highly correlated sources could potentially fall into a single component or induce cross-talk between components. The utilization of distributed sources is anticipated to alleviate this effect, although it should be confirmed by further investigations.

We used a connectivity graph to identify the leading components, in order to select those that should constitute the most relevant part of the epileptogenic network. This allowed an objective estimation of these components. Further studies involving a larger cohort of patients will enable us to assess whether the principal component highlighted on the graph is the most clinically relevant. In this context, granger causality^27^ has been used as a connectivity measure. We used the h^2^ method, initially proposed by Pijn et al. and that we used in previous publications particularly for estimating effective connectivity (‘directionality index’^28^). Additional studies are necessary to conduct comparisons among various connectivity measures and confirm the clinical relevance of h^2^ graph.

The results in our two patients are robust regarding the period of interest: similar sources are retrieved from ictal and interictal data, even though the number of components differs. This may reflect the fact that ictal and interictal activity do not involve exactly the same brains regions, with potentially different spatio-temporal dynamics. Analysing the interictal period is pertinent because of the large amount of time samples that could help the ICA estimation^29^, but it can be complicated in certain cases due to the discordance between irritative zone and seizure onset zone^30,31^. It would be interesting to compare the results with non-invasive whole-brain techniques such as MEG or high-resolution-EEG on interictal^16^ and ictal^32–34^ data, or with simultaneous ictal EEG-SEEG^35^. The two cases presented herein are epilepsies of the perisylvian/opercular region related to probable or confirmed focal cortical dysplasia. Ictal/interictal correspondence is known to be maximal in such cases^30^.

Several methods exist for localizing the activated cortex corresponding to a given distribution of potentials on the SEEG electrodes. Distributed sources are interesting as they do not require specifying the number of sources, and can potentially be used to estimate the extent of activated cortex^36^. If the source is sufficiently remote from the sensors^10^, the extent of the cortex is not too large^37^, then a dipole can also be used as a summary of activated cortex^38^. Future work on simulated data could help defining the respective advantages of different methods. The drawback of distributed sources on the cortex, as used here, is that subcortical sources, which are involved in a large proportion of patients, are not taken into account. For patients with EZN involving mesial or deep structures, it could be interesting to use a dipole scan using a grid including mesial structures. It would be also possible to use a mixed source model including cortical surfaces and deep structures^39^ as explained in https://neuroimage.usc.edu/brainstorm/Tutorials/DeepAtlas. We assumed in the source localization process that ICA removed most of the noise and used the identity matrix for the noise. Further work is needed to model the noise and test its impact on source localization.

As a methodological limitation, one could point out a lack of blinding in the preselection of epileptic components and the interpretation of the results. Nonetheless, the component selection was made on the time series and not the topographies, and the h^2^ connectivity graph allowed an automated selection of the components of interest based on the node out strength quantification, thus reducing the user-dependent bias. However, the retrospective design of the present study does not allow a blind evaluation of the pertinence of all the components of interest and we cannot exclude that discordant results would be possible in other cases. Whether such discordant results are more frequent in the cases of surgical failures should be evaluated in a prospective study. As a further limitation, the configuration of SEEG electrodes likely plays a crucial role in the quality of the source reconstruction. Thus, reconstruction can be hampered if the remaining electrodes do not encircle a source^9^, or if electrodes are too far from the sources^10^. We investigated this issue in Madrona et al.^10^ using auditory evoked potentials. We demonstrated that the auditory source could be captured at a distance of up to 40 mm from the sensors. However, this distance is impacted by the amplitude of the source. Since the epileptic spikes are likely to have higher amplitudes than evoked potentials, we presume that for spike detection, this minimal distance from the sensors could be longer. We have further shown that a minimum of three electrodes should surround the source of interest to obtained good results. Importantly, the spread of solutions can assess the confidence of a given solution and give insight on whether the spatial configuration is adequate or not. A large confidence region of the localized dipole could be used as an indicator of inadequacy of the geometrical arrangement of electrodes around the source^10^.

Finally, the approach proposed herein counterbalances the SEEG spatial undersampling, which is a classical problem of this technique. It offers the possibility to optimize the implantation strategy if a re-exploration is required, or alternatively, if adding electrodes per ongoing SEEG procedure is exceptionally planned^40^. Whether it can avoid a second implantation or limit the density of implanted electrodes (e.g. in order to diminish the risk of haemorrhagic complications in functional areas) has yet to be demonstrated. It will be important in the future to extend this study to a larger population of patients, with a perspective of validating the method in a prospective, randomized multicenter trial to measure the possible impact of this approach on the interpretation of SEEG and the consequences for the surgical strategies.

### Supplementary Information

Supplementary Figures.

## Data Availability

The datasets generated and/or analysed during the current study are not publicly available due to their clinical nature but are available from the corresponding author on reasonable request.
