# Supplementary material for: Combining independent component analysis and source localization for improving spatial sampling of stereoelectroencephalography in epilepsy
Source: Sci Rep. 2024 Feb 19;14:4071. doi: 10.1038/s41598-024-54359-4 (PMC10876572; doi:10.1038/s41598-024-54359-4)
Supplement: Supplementary file 1 — Supplementary Figures. [file 41598_2024_54359_MOESM1_ESM.docx]

**Combining Independent Component Analysis and Source Localization for Improving Spatial Sampling of Stereoelectroencephalography in epilepsy**

Samuel Medina Villalon^1,2#^, Julia Makhalova^1,2#^, Victor J. López-Madrona^2^, Elodie Garnier^2^, Jean-Michel Badier^2^, Fabrice Bartolomei^1,2*^, Christian G. Bénar^2*^

^1^APHM, Timone Hospital, Epileptology and Cerebral Rhythmology, Marseille, France

^2^Aix Marseille Univ, INSERM, INS, Inst Neurosci Syst, Marseille, France

^#,^ * Equally contributing

Supplementary Fig.1:


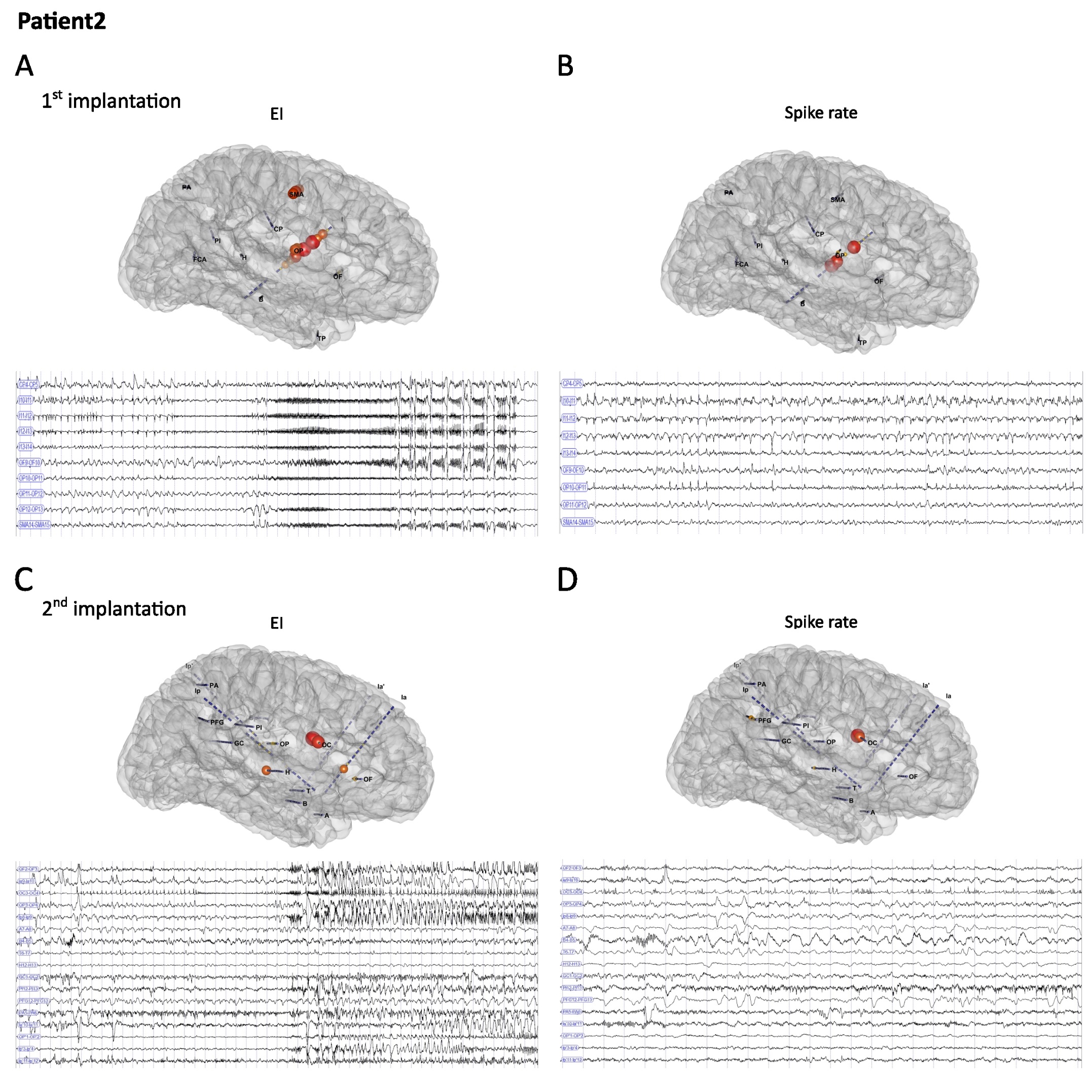


**Supplementary Fig.1**: **Illustration of the ictal and inter-ictal recordings from the 2 implantations of the patient 2.
(A)** Epileptogenicity Index (maximal EI values are represented as colored spheres in the patient’s 3D brain mesh) obtained from the first SEEG and the respective ictal SEEG time series. Seizures started by preictal spiking followed by rapid discharge over the right central operculum (OP), the insula (I2-4) and the precentral sulcus (I10-12), also involving the frontal operculum and the dorsolateral premotor cortex (SA). **(B)** Interictal data from the same SEEG. The 3D mesh of the patient’s brain showing the maximal spike rates as colored spheres and the respective interictal resting-state SEEG time series showing subcontinuous spike activity in the right central operculum (OP), the insula (I2-4) and the precentral sulcus (I10-12). **(C)** The second SEEG implantation shows the maximal epileptogenicity within the dysplasitic inferior aspect of the right precentral sulcus (OC). A habitual seizure begins with preictal spiking followed by fast discharge in this region before rapid spread throughout the right fronto-parieto-opercular and the left insulo-opercular regions. **(D)** Interictal spike rate and respective interictal time series of the 2^nd^ SEEG showing the maximal interictal activity within the dysplastic right precentral sulcus (OC3-5).

Supplementary Fig.2:


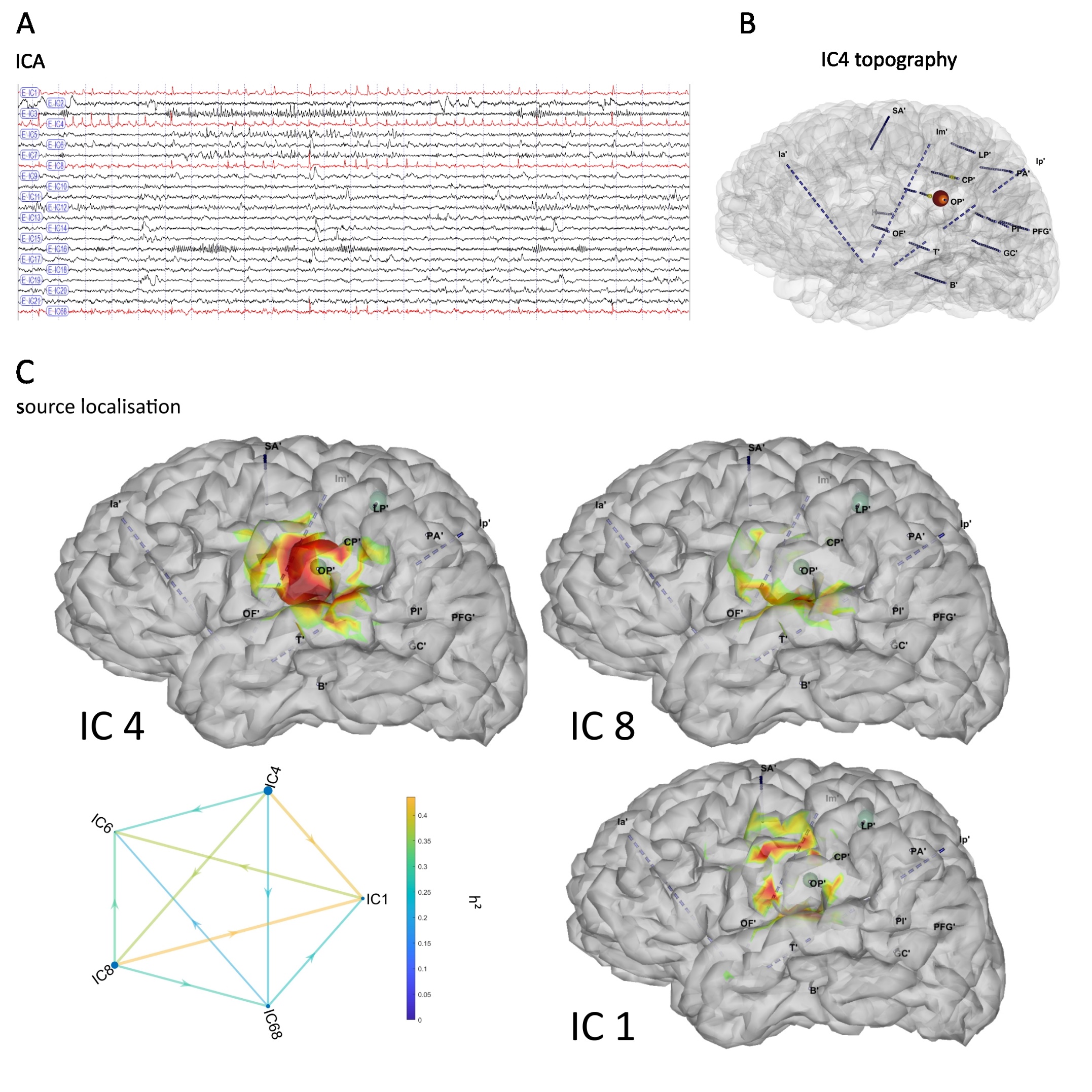


**Supplementary Fig.2**: **Illustration of the method of ICA followed by source localization on the interictal recording of Patient 1.**
**(A)** illustration of the ICA time series obtained from the monopolar SEEG recording of the first implantation. **(B)** Example of a topography (IC4) in the mesh of the patient1 represented by colored spheres. **(C)** Connectivity graph between the selected components. The links represent the mean h², the directionality is given by the directionality index and the circles the strength of each node. The 3 colored brains represent the source localization of the topographies of the 3 main components (SLoreta map threshold = 50%).

Supplementary Fig.3:


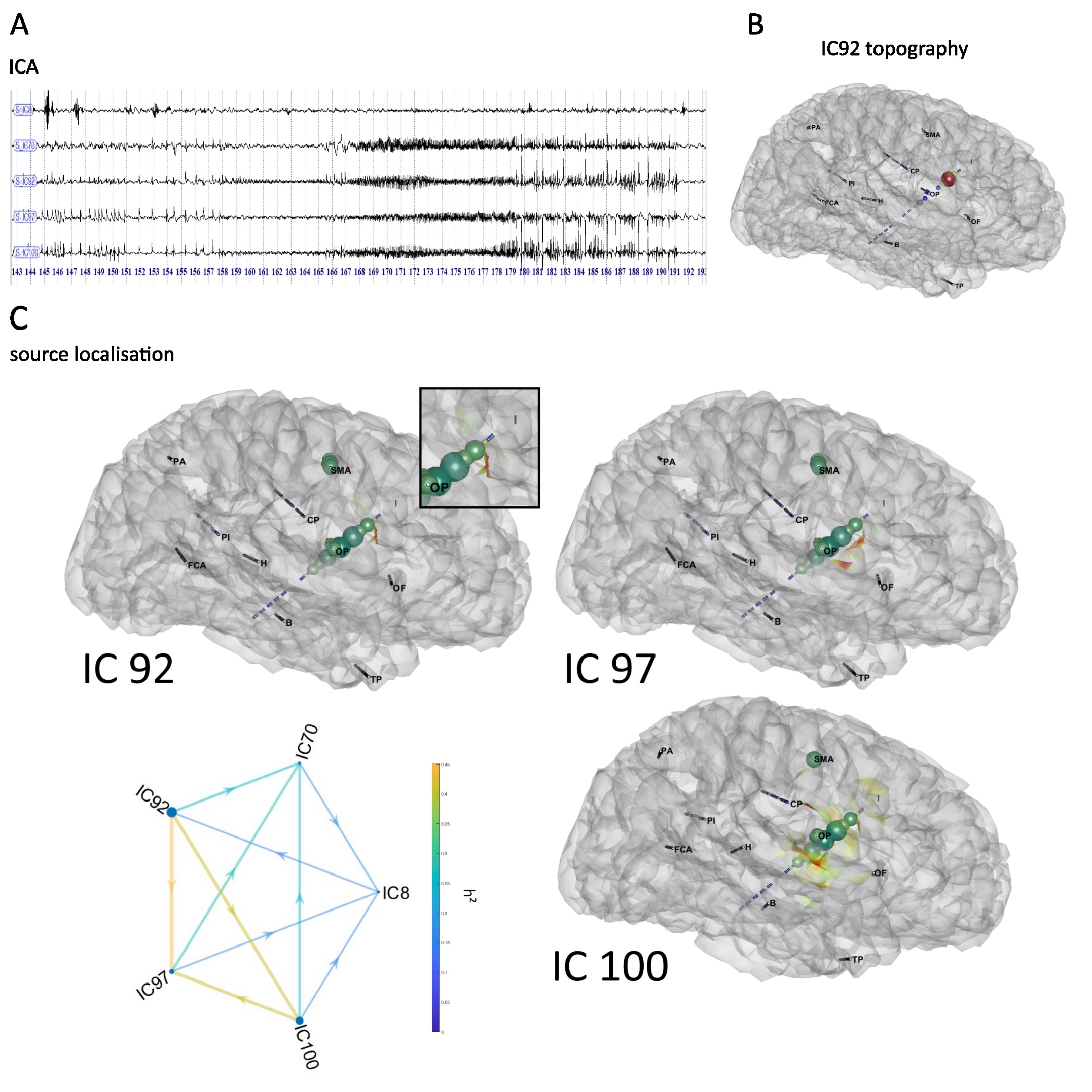


**Supplementary Fig.3:** **Illustration of the method of ICA followed by source localization on the ictal recording of Patient 2.
(A)** illustration of the ICA time series obtained from the monopolar SEEG recording of the first implantation **(B)** Example of a topography (IC92) in the mesh of the patient2 represented by colored spheres. **(C)** Connectivity graph between the selected components. The links represent the mean h², the directionality is given by the directionality index and the circles the strength of each node. The 3 colored brains represent the source localization of the topographies of the 3 main components (SLoreta map threshold = 50%).

Supplementary Fig.4:


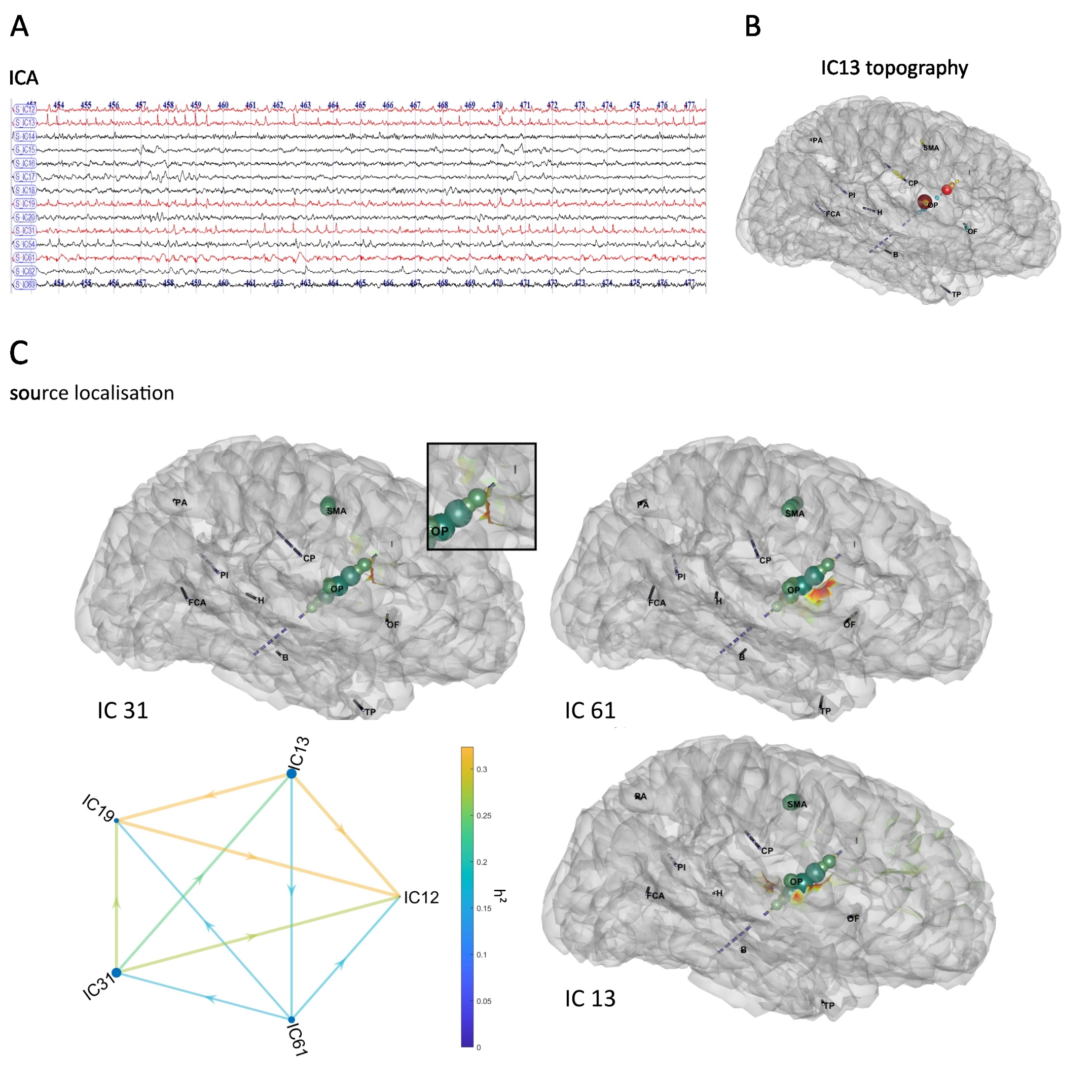


**Supplementary Fig.4:** **Illustration of the method of ICA followed by source localization on the interictal recording of Patient 2.
(A)** illustration of the ICA time series obtained from the monopolar SEEG recording of the first implantation. **(B)** Example of a topography (IC13) in the mesh of the patient2 represented by colored spheres. **(C)** Connectivity graph between the selected components. The links represent the mean h², the directionality is given by the directionality index and the circles the strength of each node. The 3 colored brains represent the source localization of the topographies of the 3 main components (SLoreta map threshold = 50%).
